# Construction and validation of a risk prediction model for clinical axillary lymph node metastasis in T1–2 breast cancer

**DOI:** 10.1038/s41598-021-04495-y

**Published:** 2022-01-13

**Authors:** Na Luo, Ying Wen, Qiongyan Zou, Dengjie Ouyang, Qitong Chen, Liyun Zeng, Hongye He, Munawar Anwar, Limeng Qu, Jingfen Ji, Wenjun Yi

**Affiliations:** 1 Department of General Surgery, The Second Xiangya Hospital, Central South University, Changsha, China; 2 Department of General Surgery, The First People’s Hospital of Changde City, Changde, China; 3 Department of General Surgery, Xiangya Hospital Central South University, Changsha, China

**Keywords:** Breast cancer, Tumour biomarkers

## Abstract

The current diagnostic technologies for assessing the axillary lymph node metastasis (ALNM) status accurately in breast cancer (BC) remain unsatisfactory. Here, we developed a diagnostic model for evaluating the ALNM status using a combination of mRNAs and the T stage of the primary tumor as a novel biomarker. We collected relevant information on T1–2 BC from public databases. An ALNM prediction model was developed by logistic regression based on the screened signatures and then internally and externally validated. Calibration curves and the area under the curve (AUC) were employed as performance metrics. The prognostic value and tumor immune infiltration of the model were also determined. An optimal diagnostic model was created using a combination of 11 mRNAs and T stage of the primary tumor and showed high discrimination, with AUCs of 0.828 and 0.746 in the training sets. AUCs of 0.671 and 0.783 were achieved in the internal validation cohorts. The mean external AUC value was 0.686 and ranged between 0.644 and 0.742. Moreover, the new model has good specificity in T1 and hormone receptor-negative/human epidermal growth factor receptor 2- negative (HR−/HER2−) BC and good sensitivity in T2 BC. In addition, the risk of ALNM and 11 mRNAs were correlated with the infiltration of M2 macrophages, as well as the prognosis of BC. This novel prediction model is a useful tool to identify the risk of ALNM in T1–2 BC patients, particularly given that it can be used to adjust surgical options in the future.

## Introduction

Breast cancer (BC) is the most common malignant tumor in women, accounting for 30% of all new cancer cases around the world^1^. The axillary lymph node (ALN) status is an important reference factor for predicting clinical outcomes in BC^2^, and it also determines the degree of axillary surgery, radiation therapy, neoadjuvant therapy and adjuvant systemic therapy. Hence, it is of clinical importance to identify axillary lymph node metastasis (ALNM) accurately.

Sentinel lymph node biopsy (SLNB) is the gold standard for evaluating the status of ALNs in patients with T1–2 BC. However, it is an invasive surgical procedure, with a false negative (FN) rate of 9.8%^3^, and approximately 50–70%^4^ of BC patients with positive sentinel lymph nodes (SLNs) do not have nonsentinel ALNM. Simultaneously, axillary lymph node dissection (ALND) or radiotherapy may be needed for patients with positive SLNs^5^. The current standard assessment methods for nodal staging in patients with T1–2 BC, such as a physical examination, ultrasound (US) or computed tomography (CT), have been shown to be less than satisfactory^6–8^.

Hence, a less invasive method is needed to evaluate the ALN status and to safely avoid the use of SLNB in patients without ALNM. There have been increasing numbers of studies with multiple prediction models for ALNM in BC that were based on several different kinds of factors, such as a combination of radiomics and kinetic curve patterns^9^, ultrasound images^10^, and miRNAs^11^. Previous studies have reported that mRNAs have been implicated in metastasis, proliferation, and apoptosis in BC^12–14^. In addition, clinical factors, such as pathological tumor size, are considered influencing factors for ALNM in BC^15^. Therefore, the construction of a model that combines mRNAs and clinicopathological factors to predict ALNM in T1–2 BC would be feasible and innovative.

In this study, we constructed a model to predict ALNM in T1–2 BC by analyzing public databases, and we also analyzed the association between the risk of ALNM and patient survival and immune cell infiltration. Therefore, we hope that this study will provide an effective and new method to predict lymphatic metastasis accurately in BC.

## Materials and methods

### Public datasets

Transcriptome profiling data (including lncRNA and mRNA data) normalized to fragments per kilobase million (FPKM) and relevant clinical information on BC from The Cancer Genome Atlas (TCGA) were downloaded. According to the specific inclusion and exclusion criteria, 465 samples in the TCGA database and 716 samples in the Gene Expression Omnibus (GEO) database were selected. All 465 patients in the TCGA were randomly divided into two independent datasets at a ratio of 7:3 based on a computer-generated random number (training dataset: 326 patients; validation dataset: 139 patients). GSE9893 served as another training dataset. The clinical characteristics of all patients are shown in Table 1 and Supplementary Table 1.

**Table 1:** Baseline characteristics of samples from the TCGA database.

Clinical features	Training set		Internal validation set		*P*-value
	N	%	N	%	
**Age**					0.213
≥ 56	180	55.2%	68	48.9%	
< 56	146	44.8%	71	51.1%	
**ER**					0.597
Negative	85	26.1%	33	23.7%	
Positive	241	73.9%	106	76.3%	
**PR**					0.214
Negative	118	36.2%	42	30.2%	
Positive	208	63.8%	97	69.8%	
**HER2**					0.230
Negative	246	75.5%	112	80.6%	
Positive	80	24.5%	27	19.4%	
**T-stage of primary tumor**					0.832
T1	97	29.8%	40	28.8%	
T2	229	70.2%	99	71.2%	
**Lymph node status**					0.993
Without metastasis	169	51.8%	72	51.8%	
With metastasis	157	48.2%	67	48.2%	
**Subtypes**					0.522
HR+/HER2−	188	57.7%	89	64.0%	
HR+/HER2+	57	17.5%	21	15.1%	
HR−/HER2+	23	7.1%	6	4.3%	
HR−/HER2−	58	17.8%	23	16.5%	
**Pathological types**					0.391
Invasive ductal carcinoma	282	86.5%	116	83.5%	
Invasive lobular carcinoma	44	13.5%	23	16.5%	

The inclusion criteria were as follows: (a) female BC patients with pathological stage T1-T2 disease; and (b) patients with a pathological diagnosis of invasive ductal carcinoma or invasive lobular carcinoma. The exclusion criteria were as follows: (a) patients with incomplete clinicopathological information, such as TX stage (the primary tumor could not be assessed), NX stage (regional lymph node involvement could not be assessed), and MX stage (the metastatic status could not be assessed) in the TNM staging system, and those with an uncertain estrogen receptor (ER), progesterone receptor (PR) and human epidermal growth factor receptor 2 (HER2) status; (b) patients who had received preoperative adjuvant therapy; and (c) patients with distant metastasis.

### Identification of differentially expressed genes (DEGs)

The “limma” package in R was utilized to select DEGs between lymph node (LN)-positive and LN-negative patients in the TCGA/GSE9893 datasets. We used the false discovery rate (FDR) < 0.05 and |log_2_FC (fold change)| > 1 as the thresholds for identifying the DEGs. A volcano diagram and a heatmap were generated using the “pheatmap” R package.

### Feature selection

We employed least absolute shrinkage and selection operator (LASSO) regression to further select the most diagnostically predictive features in the training datasets. The lymph node status served as the dependent variable, and a minimum λ was used for feature selection. Then, we used univariate logistic regression to filter the diagnostic features selected by the LASSO regression analysis. The LASSO regression analysis was performed with the “glmnet” package in R.

### Prediction model construction and performance assessment

Using a multivariate logistic regression algorithm, we constructed a risk prediction model in the training dataset. A nomogram was formulated based on the results of the multivariable analyses by integrating multiple prediction indicators. Correspondingly, the coefficient of each feature in the model and the predicted index of each patient in the training cohort were calculated. The goodness of fit between the observed value and the predicted value was tested using the Hosmer–Lemeshow test and displayed in the calibration curve. The predictive discrimination of the model was evaluated using the area under the curve (AUC) of the receiver operating characteristic (ROC) curve. Decision curve analysis (DCA) was employed to judge the clinical applicability of the nomogram by quantifying the net benefits at different threshold probabilities^16^. The prediction model was evaluated in the validation datasets. The “rms”, “ROCR” and “rmda” packages in R were applied to create the calibration curve, ROC graph and DCA curve.

### Prognostic analysis

The largest Youden index was used as the cutoff value to separate the patients into high- or low-risk groups^17^. Kaplan–Meier analysis with the log‐rank test was subsequently performed to assess the differential outcomes in overall survival (OS) or distant metastasis-free survival (DMFS) between the two groups. The Kaplan–Meier plotter (https://kmplot.com/analysis/) database^18^ was applied to analyze the difference in recurrence-free survival (RFS) according to target gene expression.

### Functional analysis and immune infiltration

We performed Gene Ontology (GO) and Kyoto Encyclopedia of Genes and Genomes (KEGG) enrichment analyses on the DEGs using the “clusterProfiler” package in R. ImmuCellAI (http://bioinfo.life.hust.edu.cn/ImmuCellAI#!/) and CIBERSORT were used to estimate tumor immune infiltration in each sample in the TCGA cohort^19,20^. Based on the “Gene” module of the TIMER2.0 database^21^ (http://timer.cistrome.org/), we further evaluated the correlation between the hub genes and the infiltration of M2 macrophages.

### Statistical analysis

Statistical analysis was performed using R software (version 4.0.2). The Wilcoxon rank‐sum test is a nonparametric statistical test mainly utilized for comparing two groups, and the Kruskal–Wallis test is suitable for comparing two or more groups. A conventional two-sided *P*-value < 0.05 was considered significant.

## Results

### Clinical characteristics of the patients

We developed a risk prediction model for clinical ALNM in T1–2 BC patients. A flow chart of the whole study design is shown in Supplementary Fig. 1. The clinical features of the patients in the training and internal validation sets in the TCGA are given in Table 1, and no differences in baseline characteristics were observed between the two groups (*P*-value > 0.05). We also summarized the relevant clinical information of the BC samples from the GEO database that met the inclusion and exclusion criteria (Supplementary Table 1).

### Development of a risk prediction model for BC

**Figure 1:**
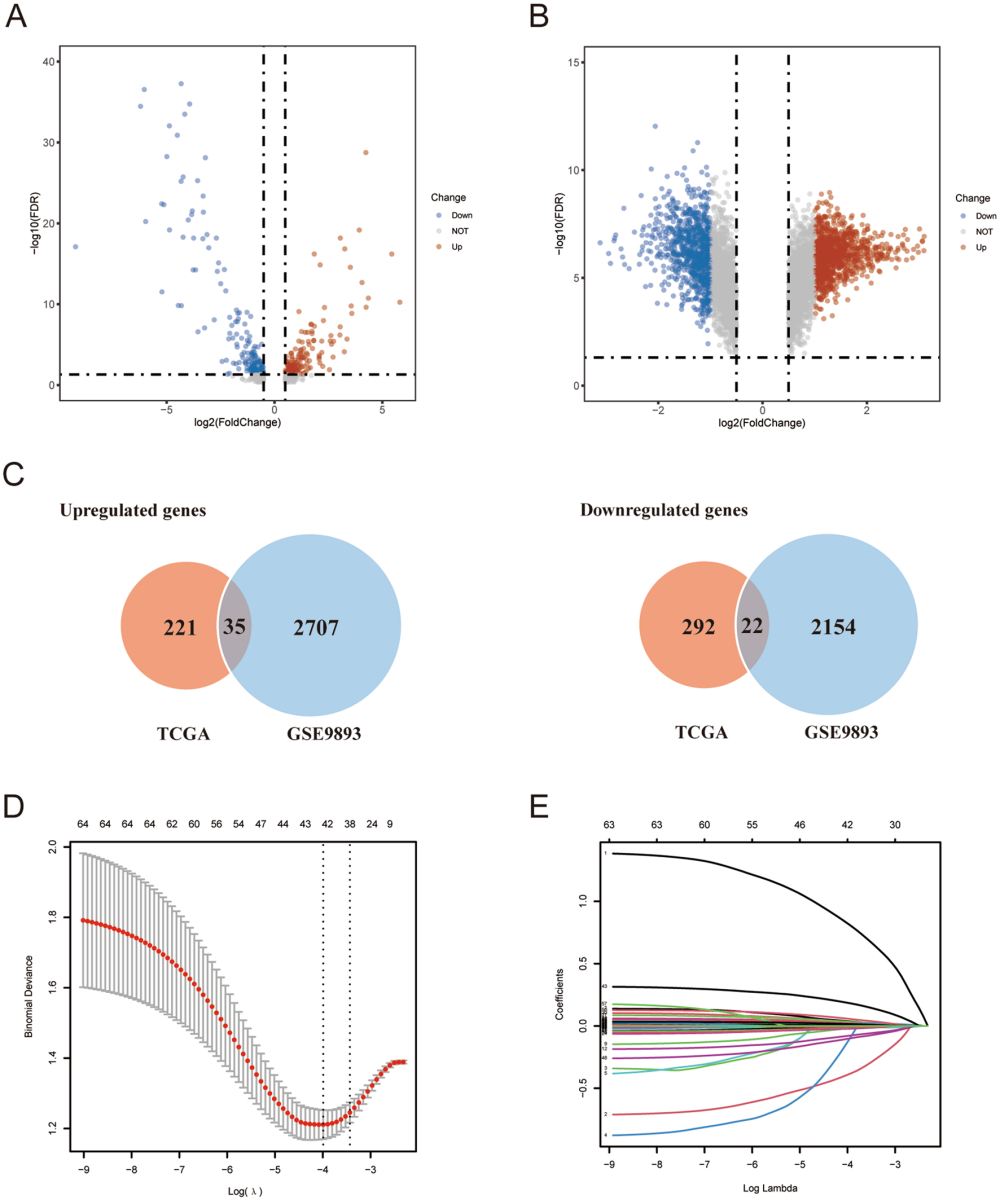
Building the risk prediction model for T1-2 invasive breast cancer. (**A**, **B**) Volcano plots of the TCGA and GSE9893 datasets; (**C**) Wayne figure of common genes between the TCGA and GSE9893 datasets; (**D**, **E**) Feature selection in the training set with the LASSO method.

Differential gene expression analysis revealed 256 upregulated and 314 downregulated genes in the TCGA training sets (Fig. 1A). However, in the GSE9893 dataset, 2742 upregulated genes and 2176 downregulated genes were found (Fig. 1B). Therefore, the results revealed 35 common upregulated genes and 22 common downregulated genes (Fig. 1C). A total of 57 mRNAs were selected as biomarker candidates and used together with clinicopathologic factors (T stage, age, ER, PR, HER2, and subtypes) to construct a diagnostic model in the training set using LASSO regression analysis (Fig. 1D,E). The heatmap shows the correlations between the expression of the 57 mRNAs and clinicopathological variables. Compared with non-axillary lymph node metastasis (NALNM) group, the expression levels of many genes were higher in the ALNM group, such as HOXB2, HOXB5, HOXB7 (Supplementary Fig. 2). In addition, the optimal value of λ in LASSO regression was 0.0185.

Subsequently, all significant factors in the univariate logistic regression analysis (Supplementary Table 2) were included in the multivariate logistic regression analysis. Finally, we constructed a risk prediction model that contains the T stage of the primary tumor and 11 genes in T1–2 BC, as shown in Supplementary Table 3.

### Nomogram construction and validation

**Figure 2:**
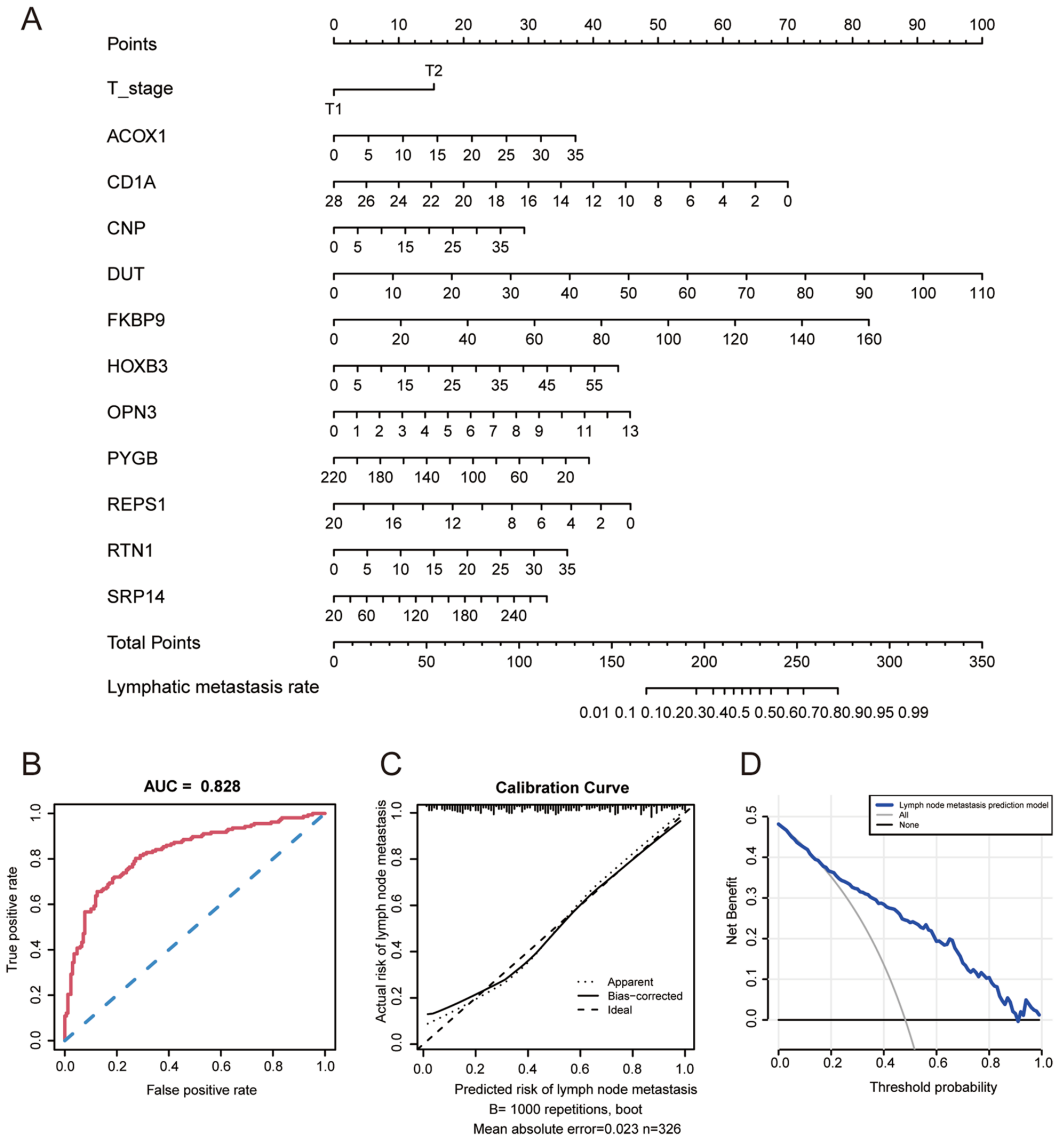
Efficacy of the risk prediction model in T1-2 invasive breast cancer. (**A**) Nomogram for the model; (**B**–**D**) ROC curve, calibration plot and decision curve analysis of the nomogram in predicting lymph node metastasis in the TCGA training sets.

These 12 features that are associated with ALNM in BC were used to construct the nomogram (Fig. 2A). This nomogram can be used to estimate the probability of ALNM through the summation of the points of each variable.

To compare the discrimination ability of the model in predicting ALNM, we conducted ROC curve analysis of T1–2 BC patients. The AUC value of the model in the training set was 0.828 (95% CI: 0.783–0.873; *P*-value < 0.001), which indicated that the model had high prediction efficacy (Fig. 2B). Furthermore, the calibration curve of the model demonstrated good agreement between the predictions and observations in the TCGA training set (Fig. 2C). The Hosmer–Lemeshow test suggested that the model had good fit (χ^2^ = 8.859; *P*-value = 0.354). Moreover, the prediction model showed a high net benefit to aid in clinical decisions for a risk probability threshold between 2 and 91% in the training set according to the DCA curve (Fig. 2D).

**Figure 3:**
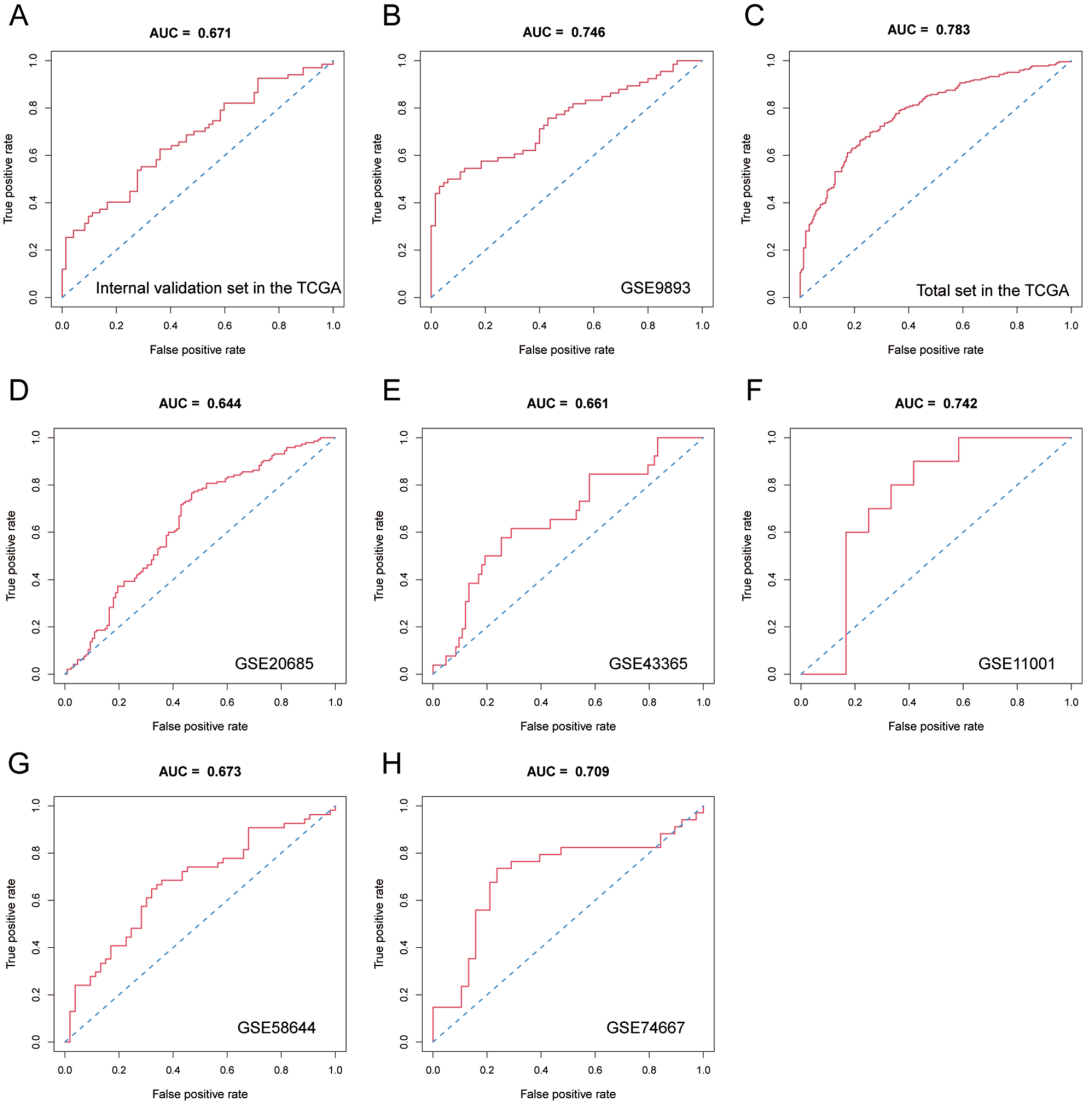
Discrimination ability of the model in the internal verification and external verification cohorts. (**A**–**C**) ROC curve analysis of the model in the internal validation cohort in the TCGA and GSE9893 and the total set in the TCGA; (**D**–**H**) ROC curve analysis of the model in the external verification cohorts, such as GSE20685, GSE43365, GSE11001, GSE58644 and GSE74667.

In the internal validation stage of the model, the AUC values were found to be 0.671, 0.746 and 0.783 (Fig. 3A–C, Supplementary Table 4). In the external validation cohort, the AUC value in the GSE11001 dataset was up to 0.742, while the AUC values in the other GEO datasets were 0.644, 0.661, 0.673, and 0.709 (Fig. 3D–H, Supplementary Table 4). The calibration curves showed great calibration of the risk prediction model in the internal and external validation cohorts (Supplementary Fig. 3).

**Figure 4:**
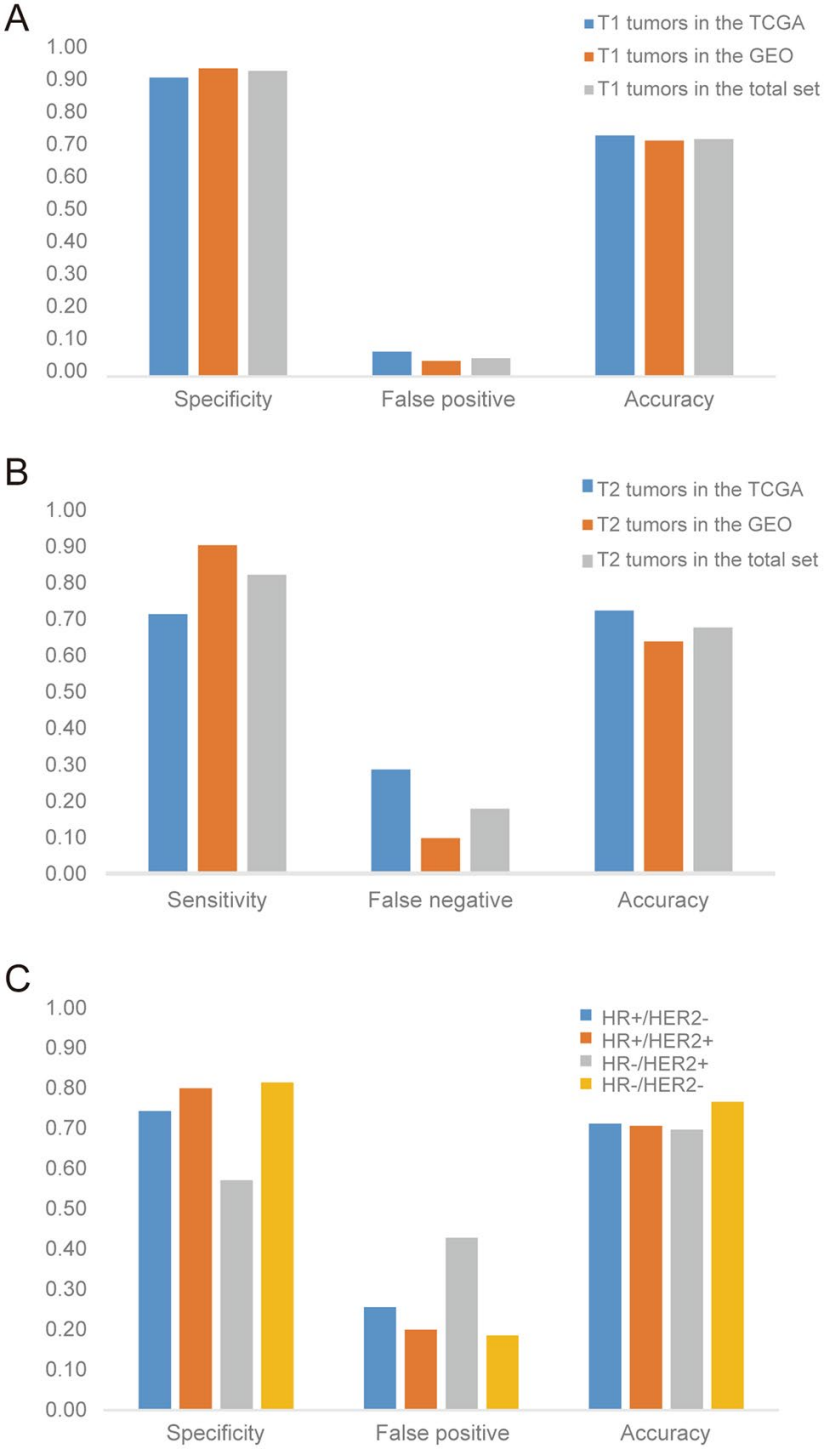
Effectiveness of the model in different T stages and different molecular types of breast cancer. (**A**, **B**) Female patients with early (T1 or T2) breast cancer; (**C**) different breast cancer molecular subtypes in the total set.

Furthermore, the specificity of the risk prediction model in predicting ALNM in T1 BC was as high as 92.3–95.1%, and the false positive rate was between 4.9 and 7.7% (Fig. 4A, Supplementary Table 5). In patients with T2 BC, the sensitivity of the model was between 71.3 and 90.3% (Fig. 4B, Supplementary Table 6). The model also had good specificity in evaluating the risk of ALNM in HR−/HER2− BC patients (Fig. 4C, Supplementary Table 7).

### The prognostic role of the risk prediction model

**Figure 5:**
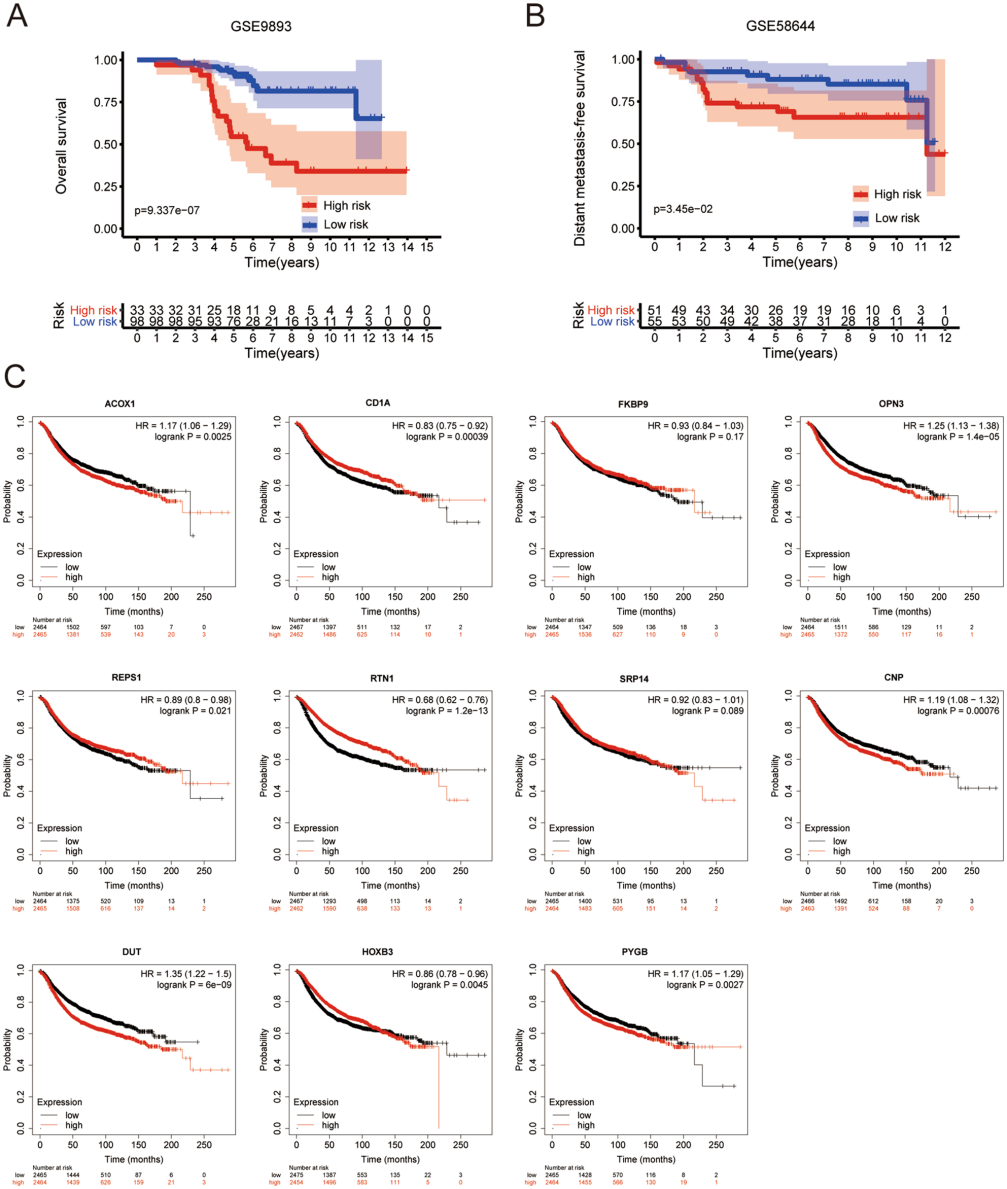
Prognostic value of the risk prediction model. (**A**) Kaplan–Meier OS curve in GSE9893; (**B**) DMFS curves for breast cancer patients in GSE58644; (**C**) Kaplan–Meier survival curves according to genes in the model.

All patients were separated into high- or low-risk groups according to the optimal cutoff value. Kaplan–Meier analyses were subsequently performed to assess the differential outcomes between the two groups. Patients in the high-risk group had worse OS (*P*-value = 9.337e−07) and DMFS (*P*-value = 3.45e−02) (Fig. 5A,B).

To further evaluate the prognostic value of genes in the risk prediction model, the RFS of BC patients was investigated with the Kaplan–Meier Plotter database. Interestingly, the expression of ACOX1, CD1A, OPN3, REPS1, RTN1, CNP, DUT, HOXB3, and PYGB was significantly associated with the prognosis of BC (*P*-value < 0.05), which was consistent with the predictive value of the model (Fig. 5C).

### Analysis of gene functions and tumor immune infiltration

**Figure 6:**
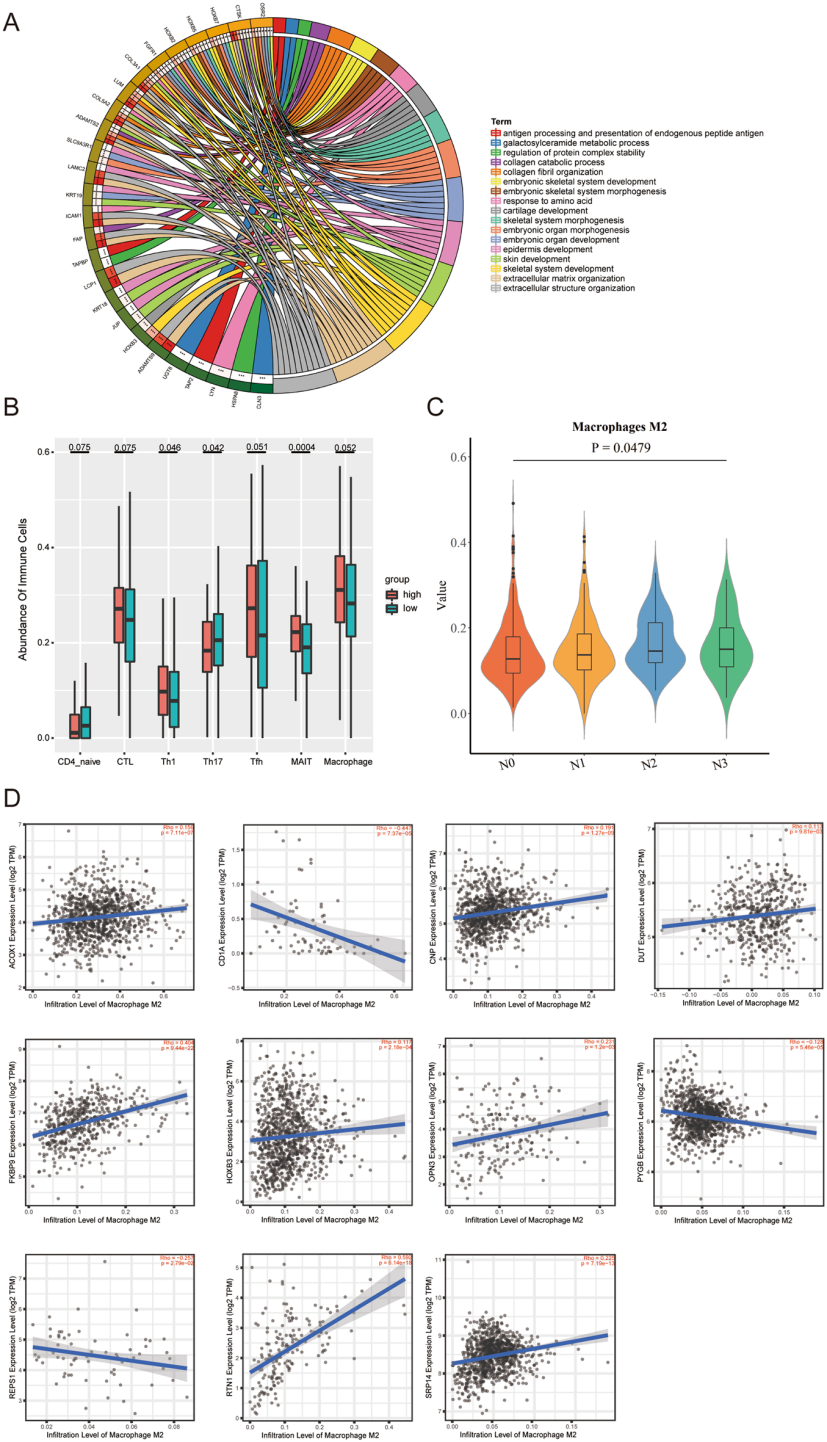
Analyses of gene function and tumor immune infiltration. (**A**) GO results revealed that the DEGs were involved in some immune-related processes; (**B**) Risk cores correlated with immunocyte infiltration in the TCGA cohort; (**C**) Lymph node stage in breast cancer correlated with M2 macrophages; (**D**) Correlation of the expression of 11 genes with M2 macrophages in breast cancer.

We analyzed the common differentially expressed genes by functional enrichment analysis, and the results indicated that the abovementioned genes mostly function in the processes of antigen processing and endogenous peptide antigen (Fig. 6A), which suggests that the common genes may be associated with tumor immune infiltration. Furthermore, we evaluated the correlation between the risk of ALNM in T1–2 BC patients and the immune infiltration level with the ImmunCellAI online resource. The results showed that the risk of ALNM was related to the infiltration of tumor immune cells such as macrophages, T helper type 1 cells (Th1), T helper type 17 cells (Th17), and cytotoxic T cells (CTLs) (Fig. 6B).

We calculated the 22 subpopulations of immune cells in 685 BC patients (according to the previous inclusion and exclusion criteria of the TCGA database, patients were re-incorporated without restricting the expression of ER,PR and HER2) by using the CIBERSORT algorithm and investigated the differences between tissues with different N stages. Surprisingly, the lymph node stage was correlated with the infiltration of M2 macrophages (*P*-value < 0.05) (Fig. 6C). Based on the TIMER2.0 database, we further explored the functions of the 11 genes in the model, all of which were associated with the infiltration of M2 macrophages (Fig. 6D).

## Discussion

ALNs are often the first and most frequent metastatic site and are the most important factor for the diagnosis and prognosis of BC^22,23^. In the current study, we developed and validated a risk prediction model to evaluate the probability of positive lymph nodes in patients with T1–2 BC to improve the ability of preoperative individualized treatment decisions in the future.

As mentioned previously, the risk prediction model incorporates 11 genes that may be related to ALNM and the T stage of the primary tumor. The results of this study showed that the risk of ALNM was positively correlated with tumor size, and similar results have been reported^15^.

To confirm the generalizability and repeatability of the established model, we used internal verification and external verification cohorts for further analysis. Generally, an AUC value greater than 0.75 indicates high accuracy, 0.75 to 0.6 indicates general accuracy, and less than 0.6 indicates low accuracy^24^. In this study, the distinguishing ability of the risk prediction model in T1–2 BC was acceptable regardless of whether it was used in either the internal or external validation cohort. Furthermore, the model had good specificity in predicting ALNM in T1 or HR−/HER2− BC patients, which means that SLNB may be omitted in patients who are classified as low risk according to this model based on the patient’s condition. In stage T2 patients, the model showed good sensitivity, suggesting that patients who are classified as high risk need to receive neoadjuvant therapy or accept ALND directly. Interestingly, although the model was designed to predict ALNM in T1–2 BC, we found that it could be used to predict patient survival, supporting the clinical and prognostic value of the model.

The expression of acyl-CoA oxidase 1 (ACOX1) is associated with brain metastasis in BC^25^. CD1a molecule (CD1A) may predict regional lymph node invasion and prognosis in BC^26^. Deoxyuridine triphosphatase (DUT) is correlated with the treatment of BC^27^. FKBP prolyl isomerase 9 (FKBP9) has been shown to be related to distant metastasis in prostate cancer^28^. The frequency of FKBP9 mutation is relatively high in BC^29^. A previous study revealed that low expression of homeobox B3 (HOXB3) was associated with a poor prognosis in hormone receptor-negative BC^30^. Glycogen phosphorylase B (PYGB) has potential applications in the prevention of BC metastasis^31^. Signal recognition particle 14 (SRP14) plays a role in OS in acute myeloid leukemia patients^32^. The expression of 2′,3′-cyclic nucleotide 3′ phosphodiesterase (CNP) correlates with glioblastoma patient survival^33^. Opsin 3 (OPN3) promotes epithelial-mesenchymal transition and tumor metastasis in lung adenocarcinoma^34^. RALBP1-associated Eps domain-containing 1 (REPS1) may be involved in neurodegeneration with brain iron accumulation^35^. The last gene, reticulon 1 (RTN1), is believed to be associated with prognosis and evolution in malignant glioma^36^. Furthermore, many genes in 57 candidate biomarkers, such as HOXB2, HOXB5, HOXB7, COL3A1, COL5A2, KRT18 and KRT19, are closely related to tumorigenesis, metastasis and invasion of cancer^37–43^.

In this study, the odds ratios (ORs) of eight genes in the risk prediction model were more than 1, suggesting that the aforementioned genes, such as ACOX1 and DUT, facilitate lymph node metastasis in BC. In addition, we found that the high expression of ACOX1, CNP, DUT and OPN3 was associated with poor survival. This finding is consistent with the hypothesis that the above genes may serve as adverse prognostic indicators of survival by affecting ALNM in BC patients. Similarly, BC patients with high CD1A or REPS1 expression had longer survival times than those with low CD1A or REPS1 expression.

Previous studies have shown that M2 macrophages play protumor roles^44^ and that the infiltration of M2 macrophages is correlated with lymph node metastasis of BC^45^. A similar result was found in this study. We found that the risk of ALNM and the 11 genes in the risk prediction model were correlated with the infiltration of M2 macrophages by bioinformatics analysis, which indicates that the above genes may affect ALNM in BC by participating in tumor immune infiltration.

In summary, we innovatively constructed a risk prediction model that contains the T stage of the primary tumor and 11 genes in T1–2 BC, although we used different cohorts for internal and external verification. However, this was a retrospective study, and further multicenter studies with larger sample sizes are needed to demonstrate its potential clinical application value in the future.

## Supplementary Information

Supplementary Figures.

Supplementary Tables.

## Data Availability

The data which used in this article are public data.
