# Supplementary material for: Construction and validation of a risk prediction model for clinical axillary lymph node metastasis in T1–2 breast cancer
Source: Sci Rep. 2022 Jan 13;12:687. doi: 10.1038/s41598-021-04495-y (PMC8758717; doi:10.1038/s41598-021-04495-y)
Supplement: Supplementary file 1 — Supplementary Figures. [file 41598_2021_4495_MOESM1_ESM.docx]

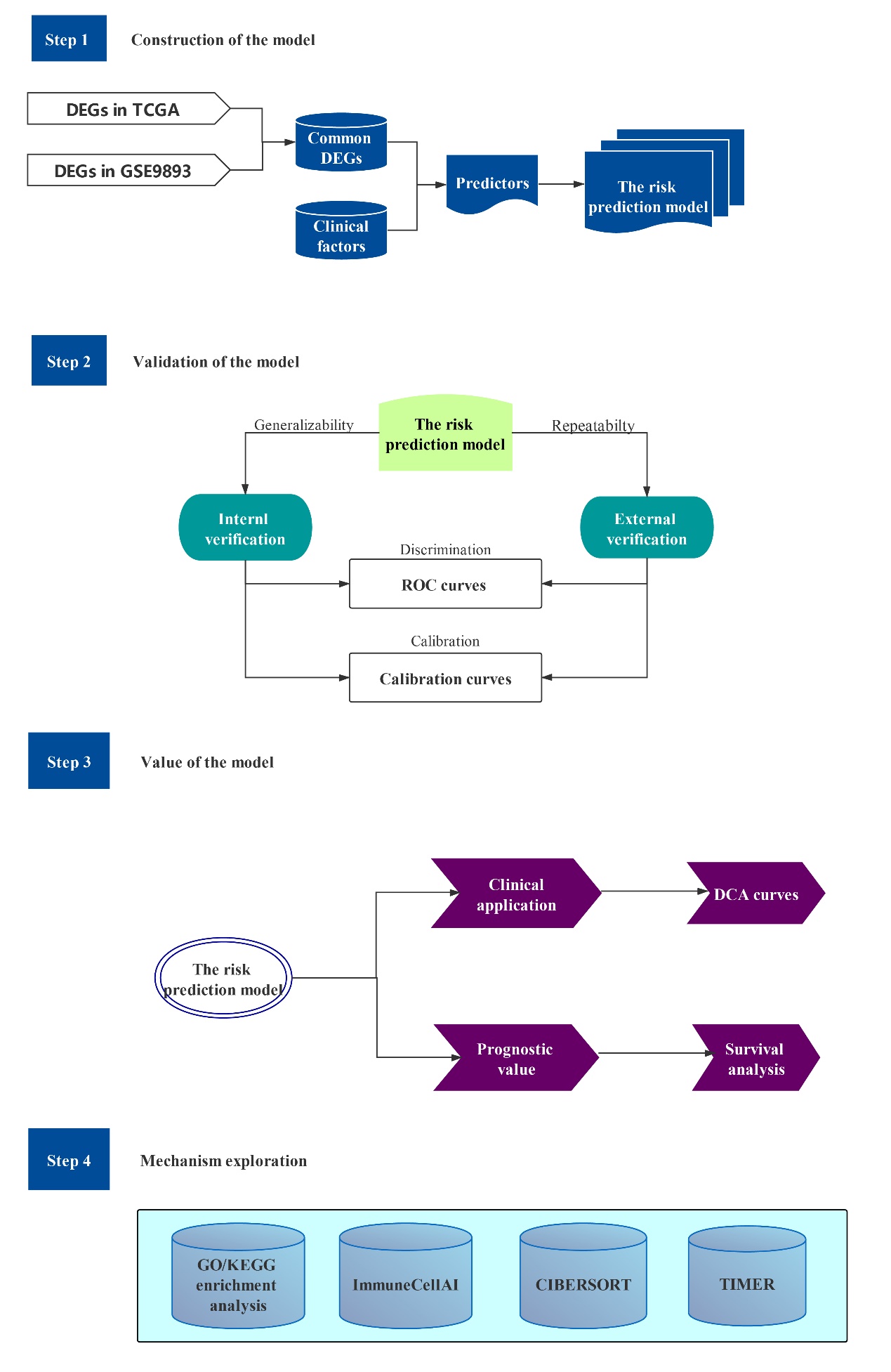


Supplementary Figure 1 Flow chart of this study.


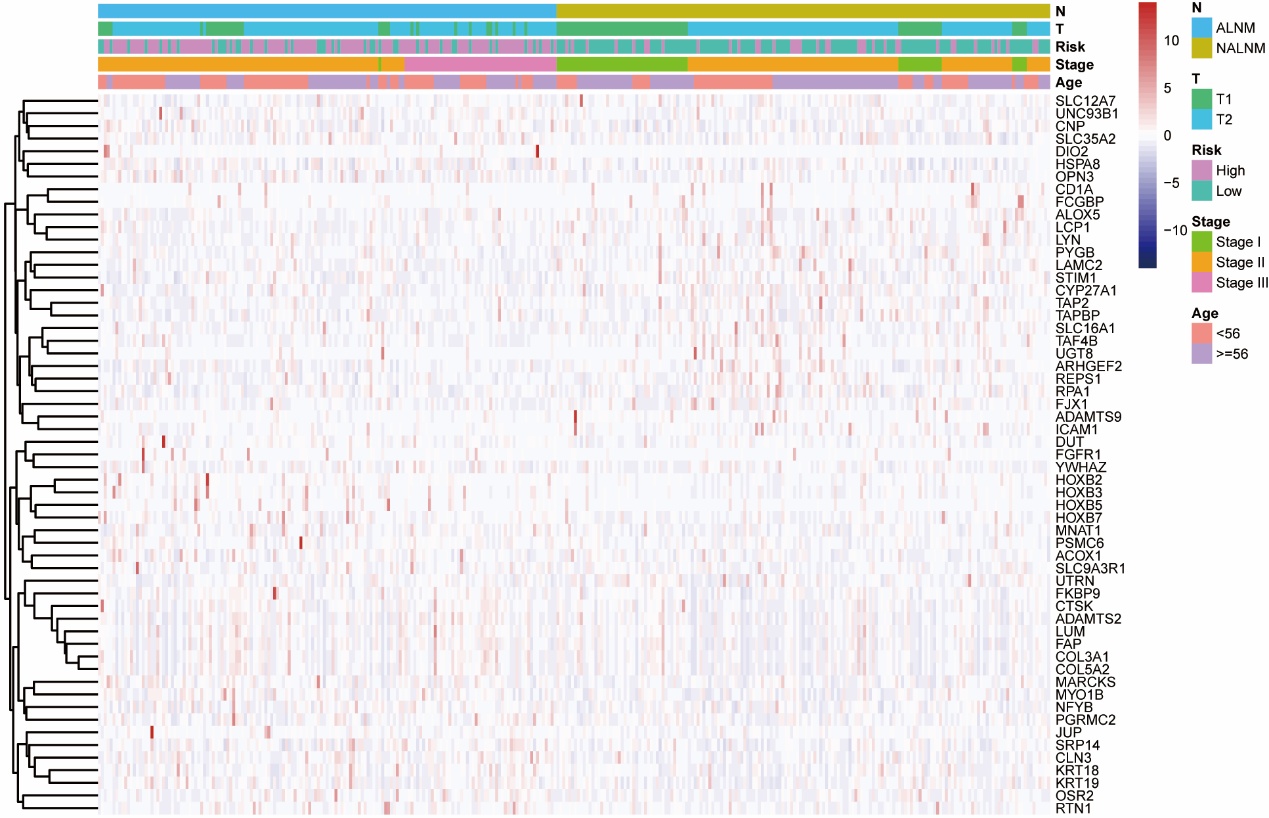


Supplementary Figure 2 The heatmap between the expression of 57 signatures and the distribution of clinicopathological variables in the non-axillary lymph node metastasis(NALNM) and ALNM patients.


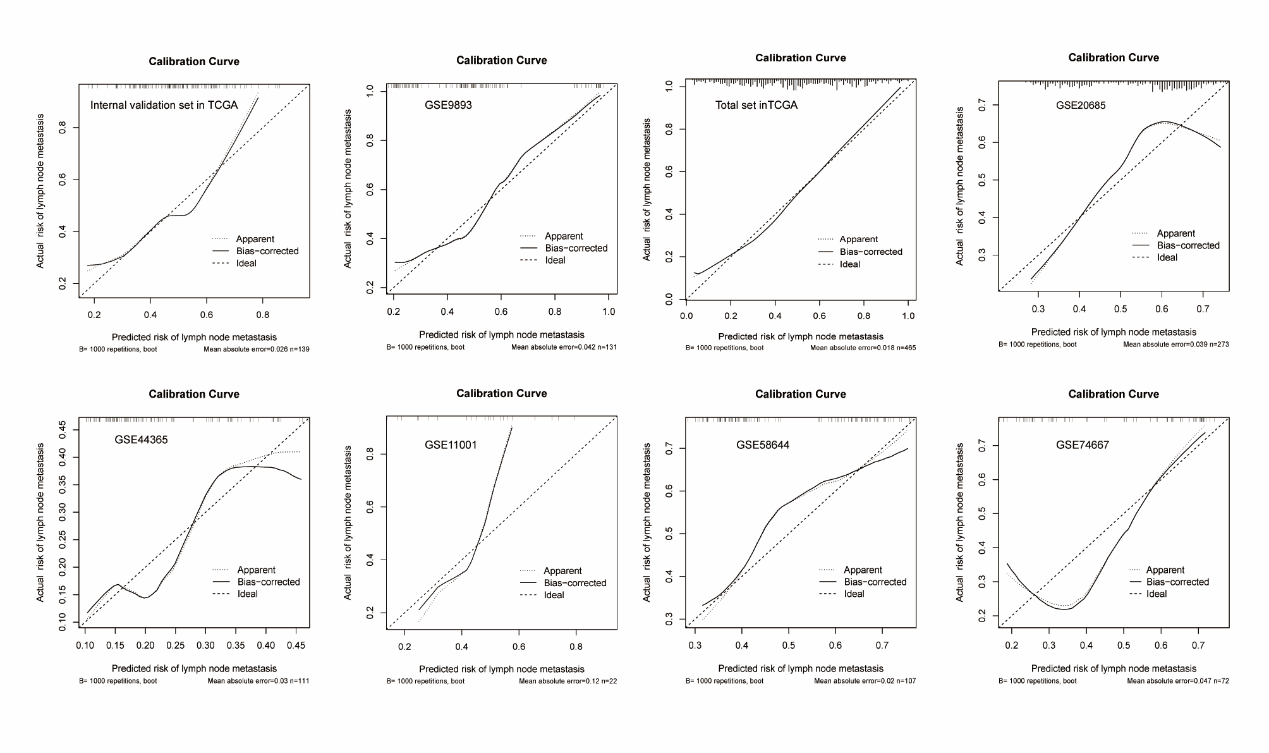


Supplementary Figure 3 Calibration curves of the risk prediction model in T1-2 invasive breast cancer.
