# Supplementary material for: Construction and validation of a risk prediction model for clinical axillary lymph node metastasis in T1–2 breast cancer
Source: Sci Rep. 2022 Jan 13;12:687. doi: 10.1038/s41598-021-04495-y (PMC8758717; doi:10.1038/s41598-021-04495-y)
Supplement: Supplementary file 2 — Supplementary Tables. [file 41598_2021_4495_MOESM2_ESM.docx]

Supplementary Table 1 Baseline characteristics of samples from the GEO database.

| Clinical features | GSE9893 | | GSE20685 | | GSE43365 | | GSE11001 | | GSE58644 | | GSE74667 | |  |
| --- | --- | --- | --- | --- | --- | --- | --- | --- | --- | --- | --- | --- | --- |
| Country | France | | China | | USA | | Germany | | Switzerland | | Sweden | |  |
| Platform | | GPL5049 | | GPL570 | | GPL570 | | GPL570 | | GPL6244 | | GPL6480 | |
| Numbers | | 131 | | 273 | | 111 | | 22 | | 107 | | 72 | |
| Age | |  | |  | |  | |  | |  | |  | |
| ≥56 | | 111 | | 63 | | 62 | | NA | | 54 | | 28 | |
| <56 | | 20 | | 210 | | 49 | | NA | | 53 | | 44 | |
| Unknown | | 0 | | NA | | 0 | | 22 | | 0 | | 0 | |
| ER | |  | |  | |  | |  | |  | |  | |
| Negative | | NA | | NA | | 18 | | 8 | | 20 | | 18 | |
| Positive | | NA | | NA | | 93 | | 14 | | 87 | | 54 | |
| Unknown | | 131 | | 273 | | 0 | | 0 | | 0 | | 0 | |
| PR | |  | |  | |  | |  | |  | |  | |
| Negative | | NA | | NA | | 34 | | 11 | | NA | | 26 | |
| Positive | | NA | | NA | | 77 | | 11 | | NA | | 46 | |
| Unknown | | 131 | | 273 | | 0 | | 0 | | 107 | | 0 | |
| HER2 | |  | |  | |  | |  | |  | |  | |
| Negative | | NA | | NA | | 96 | | 16 | | 87 | | 54 | |
| Positive | | NA | | NA | | 13 | | 6 | | 20 | | 16 | |
| Unknown | | 131 | | 273 | | 2 | | 0 | | 0 | | 2 | |
| T-stage of primary tumor | |  | |  | |  | |  | |  | |  | |
| T1 | | 51 | | 98 | | 70 | | 11 | | 61 | | 33 | |
| T2 | | 80 | | 175 | | 41 | | 11 | | 46 | | 39 | |
| Lymph node status | |  | |  | |  | |  | |  | |  | |
| Without metastasis | | 65 | | 128 | | 85 | | 12 | | 53 | | 38 | |
| With metastasis | | 66 | | 145 | | 26 | | 10 | | 54 | | 34 | |
| Subtypes | |  | |  | |  | |  | |  | |  | |
| HR+/HER2- | | NA | | NA | | 85 | | 11 | | NA | | 44 | |
| HR+/HER2+ | | NA | | NA | | 7 | | 3 | | NA | | 11 | |
| HR-/HER2+ | | NA | | NA | | 6 | | 3 | | NA | | 5 | |
| HR-/HER2- | | NA | | NA | | 11 | | 5 | | NA | | 10 | |
| Unknown | | 131 | | 273 | | 2 | | 0 | | 107 | | 2 | |

Supplementary Table 2 Univariate analysis of candidate factors in the risk prediction model.

| Factors | *P*-value |
| --- | --- |
| Subtype | 0.020 |
| T Stage | <0.001 |
| Age at the time of diagnosis | 0.012 |
| Progesterone Receptor | 0.787 |
| ACOX1 | 0.016 |
| ADAMTS2 | 0.025 |
| ADAMTS9 | 0.044 |
| ARHGEF2 | 0.002 |
| CD1A | 0.022 |
| CNP | 0.040 |
| CTSK | 0.030 |
| DIO2 | 0.004 |
| DUT | 0.033 |
| FGFR1 | 0.069 |
| FJX1 | 0.024 |
| FKBP9 | 0.018 |
| HOXB3 | 0.004 |
| HOXB5 | 0.004 |
| HSPA8 | 0.028 |
| ICAM1 | 0.017 |
| JUP | 0.051 |
| KRT19 | 0.019 |
| LAMC2 | 0.025 |
| LCP1 | 0.030 |
| MARCKS | 0.012 |
| MYO1B | 0.004 |
| NFYB | 0.039 |
| OPN3 | 0.003 |
| OSR2 | 0.038 |
| PGRMC2 | 0.016 |
| PYGB | 0.004 |
| REPS1 | 0.008 |
| RPA1 | 0.025 |
| RTN1 | 0.030 |
| SLC12A7 | 0.045 |
| SLC35A2 | 0.046 |
| SRP14 | 0.004 |
| STIM1 | 0.024 |
| TAPBP | 0.023 |
| UNC93B1 | 0.043 |
| UTRN | 0.035 |
| YWHAZ | 0.047 |

Supplementary Table 3 Multivariate analysis of candidate factors in the risk prediction model.

| Factors | Coefficient（B） | Std.Error | *P*-value | OR | Lower 95% CI | Upper 95% CI |
| --- | --- | --- | --- | --- | --- | --- |
| T Stage | 1.370 | 0.315 | <0.001 | 3.934 | 2.153 | 7.417 |
| ACOX1 | 0.095 | 0.037 | 0.011 | 1.099 | 1.025 | 1.187 |
| CD1A | -0.222 | 0.075 | 0.003 | 0.801 | 0.674 | 0.905 |
| CNP | 0.065 | 0.026 | 0.011 | 1.067 | 1.016 | 1.124 |
| DUT | 0.081 | 0.029 | 0.005 | 1.084 | 1.030 | 1.150 |
| FKBP9 | 0.046 | 0.016 | 0.005 | 1.047 | 1.015 | 1.082 |
| HOXB3 | 0.065 | 0.023 | 0.006 | 1.067 | 1.022 | 1.123 |
| OPN3 | 0.312 | 0.078 | <0.001 | 1.366 | 1.179 | 1.605 |
| PYGB | -0.016 | 0.006 | 0.013 | 0.984 | 0.971 | 0.996 |
| REPS1 | -0.203 | 0.065 | 0.002 | 0.816 | 0.713 | 0.920 |
| RTN1 | 0.091 | 0.032 | 0.004 | 1.096 | 1.033 | 1.172 |
| SRP14 | 0.011 | 0.004 | 0.011 | 1.011 | 1.003 | 1.020 |
| Constants | -6.021 | 1.748 | <0.001 | / | / | / |

Note:(Standard Error,Std.Error);(Confidence interval,CI)

Supplementary Table 4 AUC values in the internal and external validation datasets.

| Datasets | AUC | 95%CI | *P*-value |
| --- | --- | --- | --- |
| Internal validation set in the TCGA | 0.671 | 0.582–0.760 | <0.001 |
| Total set in the TCGA | 0.783 | 0.741–0.824 | <0.001 |
| GSE9893 | 0.746 | 0.662–0.830 | <0.001 |
| GSE11001 | 0.742 | 0.520–0.963 | 0.056 |
| GSE20685 | 0.644 | 0.577–0.710 | <0.001 |
| GSE43365 | 0.661 | 0.540–0.781 | 0.014 |
| GSE58644 | 0.673 | 0.571–0.775 | 0.002 |
| GSE74667 | 0.709 | 0.581–0.837 | 0.002 |

Supplementary Table 5 Effectiveness of the model in T1 breast cancer.

| Datasets | Sensitivity | False negative | Specificity | False positive | Accuracy |
| --- | --- | --- | --- | --- | --- |
| T1 tumors in the TCGA | 0.391 | 0.609 | 0.923 | 0.077 | 0.745 |
| T1 tumors in the GEO | 0.214 | 0.786 | 0.951 | 0.049 | 0.728 |
| T1 tumors in the total set | 0.271 | 0.729 | 0.943 | 0.057 | 0.733 |

Supplementary Table 6 Effectiveness of the model in T2 breast cancer.

| Datasets | Sensitivity | False negative | Specificity | False positive | Accuracy |
| --- | --- | --- | --- | --- | --- |
| T2 tumors in the TCGA | 0.713 | 0.287 | 0.733 | 0.267 | 0.723 |
| T2 tumors in the GEO | 0.903 | 0.097 | 0.232 | 0.768 | 0.638 |
| T2 tumors in the total set | 0.822 | 0.178 | 0.479 | 0.521 | 0.676 |

Supplementary Table 7 Effectiveness of the model on the total set of different subtypes of breast cancer.

| Subtypes | Sensitivity | False negative | Specificity | False positive | Accuracy |
| --- | --- | --- | --- | --- | --- |
| HR+/HER2- | 0.670 | 0.330 | 0.744 | 0.256 | 0.712 |
| HR+/HER2+ | 0.612 | 0.388 | 0.800 | 0.200 | 0.707 |
| HR-/HER2+ | 0.759 | 0.241 | 0.571 | 0.429 | 0.698 |
| HR-/HER2- | 0.676 | 0.324 | 0.814 | 0.186 | 0.766 |
